# The cost-effectiveness of hypertonic saline inhalations for infant bronchiolitis

**DOI:** 10.1186/s12913-020-05814-1

**Published:** 2020-11-02

**Authors:** Jefferson Antonio Buendía, Ranniery Acuña-Cordero

**Affiliations:** 1 Grupo de Investigación en Farmacología y Toxicología (INFARTO). Departamento de Farmacología y Toxicología, Facultad de Medicina, Universidad de Antioquia, Carrera 51D #62-29, Medellín, Colombia; 2 Departamento de Neumología Pediátrica, Hospital Militar Central, Departamento de Pediatría, Facultad de Medicina, Universidad Militar Nueva Granada, Bogotá, Colombia

**Keywords:** Bronchiolitis, Cost- effectiveness, Nebulization

## Abstract

**Background:**

Pharmacological treatment for bronchiolitis is primarily supportive because bronchodilators, steroids, and antibiotics, show little benefit. Clinical studies have suggested that nebulized 3% hypertonic solution is useful for infants with bronchiolitis. This study aims to evaluate the cost-effectiveness of the HS inhalations in infant bronchiolitis in a tropical country.

**Methods:**

Decision tree analysis was used to calculate the expected costs and QALYs. All cost and use of resources were collected directly from medical invoices of 193 patient hospitalized with diagnosis of bronchiolitis in tertiary centers, of Rionegro, Colombia. The utility values applied to QALYs calculations were collected from the literature. The economic analysis was carried out from a societal perspective.

**Results:**

The model showed that nebulized 3% hypertonic solution, was associated with lower total cost than controls (US $200vs US $240 average cost per patient), and higher QALYs (0.92 vs 0.91 average per patient); showing dominance. A position of dominance negates the need to calculate an incremental cost-effectiveness ratio.

**Conclusion:**

The nebulized 3% hypertonic solution was cost-effective in the inpatient treatment of infant bronchiolitis. Our study provides evidence that should be used by decision-makers to improve clinical practice guidelines and should be replicated to validate their results in other tropical countries.

## Background

Bronchiolitis is the primary reason for the hospitalization of infants in developed and developing countries [1]. Their mortality ranges from 0.2 to 7% [1, 2]. Morbidity and mortality mostly occur in children younger than 6 months with underlying pulmonary or cardiac disease, and those an immune deficiency [2]. In Colombia, the most common etiologic agent is the respiratory syncytial virus (RSV) [3] . In 2019, 260,873 years of life (IC 95% 208,180–347,023) were lost due to (RSV) - bronchiolitis in Colombian children under 2 years. The estimated rate was 20 DALYs / 1000 person-year (95% CI 16–27) [4]. Bronchiolitis in children under age two years, generates more years of life lost, in Colombia, than cervical cancer between 45 and 59 years (1.6 DALYs per 1000 inhabitants), epilepsy between 30 and 44 years (1 DALYs per 1000 inhabitants) and leukemia in children between 5 and 14 years (1 DALYs per 1000 inhabitants) [4]. This due, partially, that in our country, almost 60% of patients with bronchiolitis have severe respiratory distress [3]. This generates also, a high economic burden to our public health system, especially for the inappropriate use of diagnostic test and treatment in bronchiolitis. Indeed, almost 35% of the cost per patient/day was attributable to drugs, imaging tests, chest therapy, and laboratory tests that have no evidence or support in most clinical practice guidelines [5].

The pharmacological treatment for bronchiolitis is primarily supportive because bronchodilators, steroids, and antibiotics, had little benefit [6]. One of the multiple treatments studied is 3% hypertonic solution (HS). HS absorbs water from the submucosa and improving mucociliary function [7]. A recent metanalysis shows that infants treated with 3% HS presented shorter durations of hospitalization compared with normal saline (WMD = − 0.43; 95% CI = − 0.70, − 0.15) [8]. Also, Zhang et al. in other meta-analysis showed a 16% reduction in the risk of hospitalization among patients treated with HS compared to NS (risk ratio [RR]: 0.84, 95% confidence interval [CI]: 0.71–0.98, *P* = 0.03) [9].

In Colombia, most of treatments, diagnostic test and other direct cost of bronchiolitis are covered by the health system. However, due to the high burden of disease in our population, there is a critical need for studies to optimize the use of resources in bronchiolitis. This is essential for making informed decisions on resource allocation, especially in tropical countries where these resources are always scarce. In this sense, respect to HS, a recent study in Finland shows that the HS inhalations were cost-effective in the outpatient treatment of infant bronchiolitis [10]. However, there are limitations in transferring the results of economic evaluations from one country to another, especially when it comes to developed countries, given the differences in costs of health technologies, coverage policies, and willingness to pay. In this study, we used decision analysis to evaluate the cost-effectiveness of the HS inhalations in infant bronchiolitis compared to the NS inhalations or no inhalations in a tropical country.

## Methods

### Design

This study evaluated the cost-effectiveness of the HS (3–7%) inhalations for infant bronchiolitis versus or standard treatment without HS inhalations (controls). The main effectiveness outcome was the quality-adjusted life years (QALYs). The analysis was carried out from a societal perspective (included direct and indirect costs). The analytic horizon was an acute episode of bronchiolitis. Given the short time horizon, no type of discount to costs or results was applied.

### Economic model

**Fig. 1:**
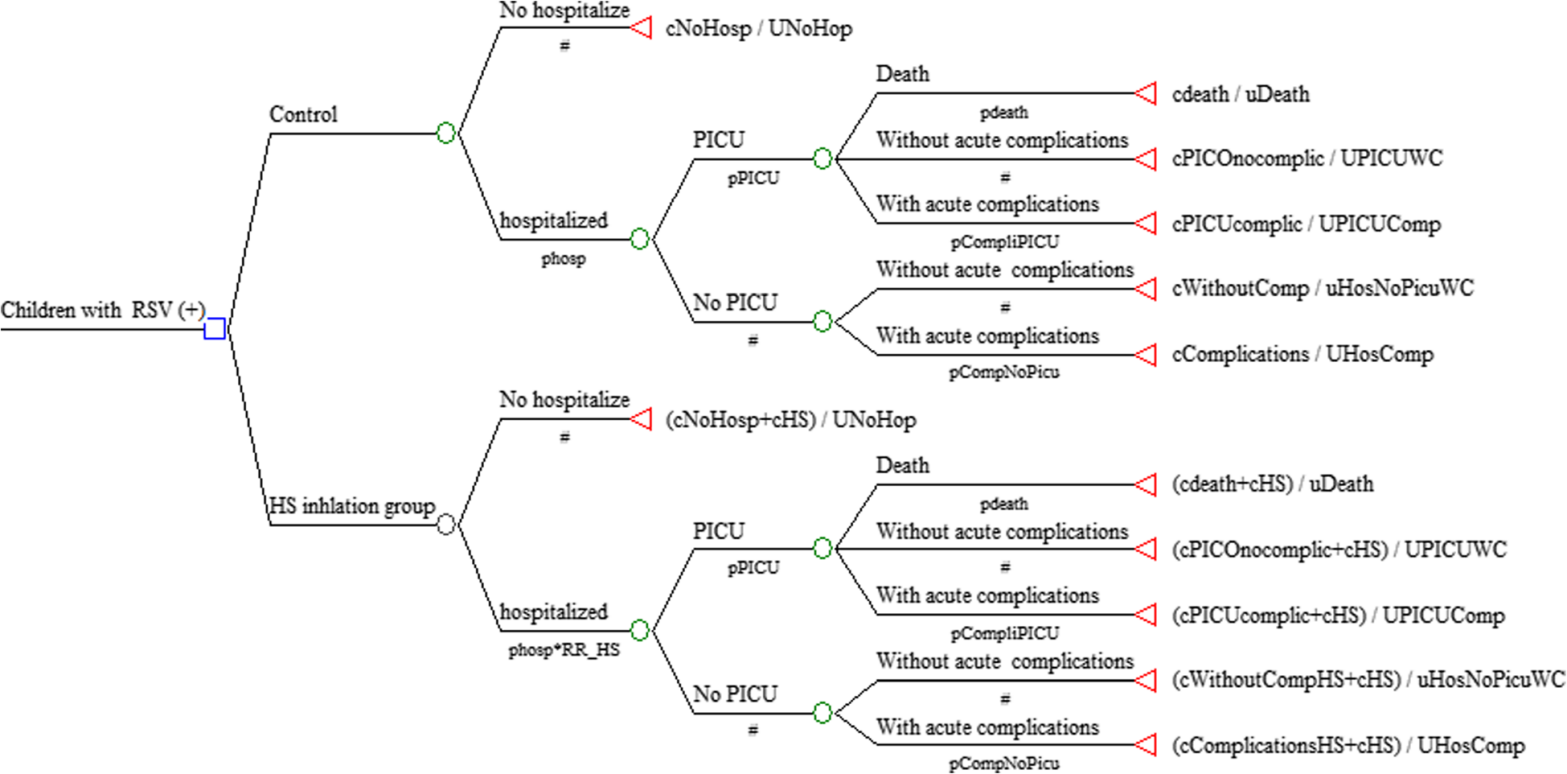
Decision tree model with probabilities estimated by outcome

A decision tree model was constructed to estimate de cost and effectiveness of episodes of bronchiolitis (Fig. 1). We defined the following outcomes according to the natural history of bronchiolitis: death, hospitalization with or without acute complications, PICU admission with or without acute complications. Among the acute complications were included: pneumonia, atelectasis, sepsis, pleural effusions, and pneumothoraxes [11]. The study protocol was reviewed and approved by the Institutional Review Board of the University of Antioquia (No 18/2015).

### Model parameters and data sources

To estimate the probabilities and utilities of the model (see Table 1), we performed a systematic review of systematic reviews or RCTs published or observational studies up to January, 2020. In order to identify potentially relevant studies were made searches of computerized databases (MEDLINE, CENTRAL, LILACS, and CINAHL); using the following search strategy: (3% hypertonic solution) AND (Bronchiolitis OR Bronchiolitis, Viral), limited with the terms children OR child OR pediatric OR adolescents OR infants OR preschoolers). No language restrictions were applied. To be included in the model, the studies had to be RCTs of parallel-group or cross-over design, systematic reviews or RCTs published or observational studies include children between 2 and 18 years of age. Also were included others observational clinical obtained during the review of references cited in published literature. The computerized search yield 1021 citations and a total of 75 studies were examined. Finally, the information was extracted for the construction of the economic model of 10 studies that included patients less than 18 years of age, with bronchiolitis, and with use of HS [12–19]. Information regarding the effect of HS was extracted from a recent meta-analysis [10, 12] which compared HS nebulization versus normal saline inhalations or standard treatment without HS nebulization (see Table 1).

**Table 1:** Model inputs: morbidity probabilities used in base case and sensitivity analyses

Model input	Base case value	SA range for one-way sensitivity analyses	Source
**Probability**			
Hospitalization	0,26	0,01-0,41	[12]
PICU, given hospitalization	0,07	0,06-0,18	[12]
Mortality, given PICU admission	0,009	0,00–0,06	[13]
Acute complications, given hospitalization	0,13	0,10–0,20	[14]
Acute complications, given PICU admission	0,15	0,15-0,53	[15]
**Utility**			
No hospitalization	0,95	1,00–0,76	[20–23]
Hospitalization without acute complications	0,88	1,00–0,70	[24, 25]
Hospitalization with acute complications	0,59	0,70–0,47	[24, 25]
PICU without acute complications	0,73	0,87-0,58	[24, 25]
PICU with acute complications	0,51	0,60–0,40	[24, 25]
**HS effectiveness**			
Reduction of hospitalization	0,80	0,67-0,96	[11]
Reduction of LOS	-0,55	-0,15-0,96	[11]

### Cost analysis

All cost and use of resources were collected directly from medical invoices of 193 patient hospitalized with diagnosis of bronchiolitis, in tertiary centers, of Rionegro, Colombia from January 2015 to December 2016(see Table 2). Details of this cost study have been previously published [26]. Briefly, the direct costs considered in the analysis include medical consultation at emergency room, specialist referrals, chest physiotherapy, RSV isolation, x-ray, oxygen, nebulization, corticosteroids, bronchodilators, medical devices, hotel services at intensive care unit, hotel services in general medical ward. We use US dollars (Currency rate: US$ 1.00 = COP$ 3000) [26] to express all costs in the study. For the valuation of the indirect costs associated with the loss of productivity of parents, the human capital method was used. The cost-opportunity of the productivity loss at the workplace and the caregiver was assessed based on the minimum wage without including the transportation assistance for the year 2019 (U$ 229.81 per month) [27]. Because all patients with bronchiolitis included were children, we assumed that at least one family, member accompanies the patient permanently during hospitalization, since pediatric hospitals in the country usually allow only one companion per patient in the hospital. The cost associated with transportation and food, was assumed to correspond to 50% of minimum wage per day.

**Table 2:** Cost used in base case and sensitivity analyses

Model input	Base case value	SA range for one-way sensitivity analyses	Distribution
**Intervention cost**			
HS cost per patient day	3,83	3,81-3,84	γ (SD:0,05)
**Hospitalization cost**			
Daily cost in paediatric ward	48.82	47,64 50.00	γ (SD:3,20)
Hospital length of stay (days)	5,8	4,00-6,01	γ (SD:2,03)
**PICU related cost**			
Daily cost in PICU	327,35	326,26–328-43	γ (SD:5,49)
PICU lenght of stay (days)	10	9,01-15,05	γ (SD:3,08)
**Emergency visit prior hospitalization cost**			
Daily cost of emergency ward	12,83	12,19-13,46	γ (SD:3,20)
**Direct medical cost per patient-day**			
Specialist referrals	10,67	10,31-11,01	γ (SD:1,72)
Chest physiotherapy	5,15	4,90-5,39	γ (SD:1,23)
Chest radiography	2,84	2,70-2,98	γ (SD:0,73)
Others diagnostic imaging	0,01	0,0-0,022	γ (SD:0,08)
Complete blood cell counts	1,12	1,05-1,17	γ (SD:0,28)
RSV test	2,71	2,83-3,03	γ (SD:2,72)
Other laboratory tests	4,40	4,23-4,47	γ (SD:0,37)
Oxygen	1,37	1,28-1,45	γ (SD:0,41)
Nebulization	16,23	1,28-1,45	γ (SD:4,52)
LEV	1,10	1,07-1,13	γ (SD:0,16)
Antibiotics systemics	1,21	1,11-1,30	γ (SD:0,49)
Systemic o Inhaled Corticosteroids	0,08	0,0-0,90	γ (SD:4,18)
Bronchodilators	0,04	0,03-0,04	γ (SD:0,02)
Other drugs	0,65	0,60–0,68	γ (SD:0,04)
Medical devices	10,24	9,71-10,76	γ (SD:2,66)
**Indirect cost patient-day**	17,24	16.38–18,07	γ (SD:4,30)

### Utilities

The utility values applied to QALYs calculations were collected from the literature. Baseline utility value for hospitalization was 0.95 [20, 28–30], whereas a 0.88 utility value was used for PICU, given hospitalization [21, 22], 0.59 for hospitalization with acute complications, and 0.5 for PICU with acute complications [23, 24]. The number of QALYs was calculated as the utility value given to a particular health state multiplied by length of time spent in that state. Given that they are utilities extracted from studies in populations other than the Colombian, a range was used for the sensitivity analysis of one way and probabilistic of more or less 20% the value of the utility.

### Sensitivity analyses

The robustness of the economic model was evaluated with one-way sensitivity analyses and probabilistic sensitivity analysis according to the recommendation of consolidated health economics evaluation reporting Standards (35). Probabilistic sensitivity analysis was run by randomly sampling from each of the parameter distributions (beta distribution in the case of relative risk, and utilities, Dirichlet distribution for multinomial data in the case of transition probabilities, and gamma distribution in the case of costs). A second-order Monte Carlo simulation were used to estimated the expected costs and QALYs of the model. Net monetary benefit was calculated by multiplying effect by societal willingness to pay and subtracting cost, with a willingness to pay set at a ratio of US$ 20,000 per QALYS. Microsoft Exel®was used in all analyzes.

## Results

The model showed that HS, was associated with lower cost than controls (US $200 vs US $240 average cost per patient), and higher QALYs (0.92 vs. 0.91 average per patient); showing dominance. A position of dominance negates the need to calculate an incremental cost-effectiveness ratio (Table 3).

**Table 3:** Cost- effectiveness of HS inhalation vs Control group

Strategy	Cost	difference	QUALYs	difference	C/E	Marg C/E
HS inhlation group	220.8		0.92		238.95	
Control	268.2	47.4	0.91	−0.004	291.84	(Dominated)

### Sensitivity analysis

One-way sensitivity analyses of parameters in the model showed that the cost-effectiveness of HS was sensitive to the probability of hospitalization. In threshold analysis, the expected cost per patient was lower in the control group only when the probability of hospitalization was equal or less than 0.022. With higher values, the expected cost of the HS group is always less than that of the control group.

**Fig. 2:**
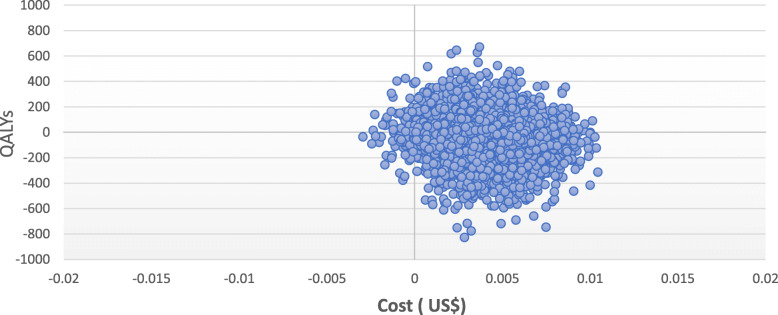
Cost-effectiveness plane

The results of probabilistic sensitivity analysis are graphically represented in the cost-effectiveness plane, Fig. 2. 61.94% of simulations were graphed in quadrant 2 (lower cost, high QALYSs), and 37.11% in quadrant 1 of this plane (high cost, high QALYSs). The 95% CI for the cost per patient treated with HS, and with controls were US$ 178 to US$ 222 and US$ 213 to US$ 267. The 95% CI for QALYs per patient was 0.922 to 0.924 and 0.918 to 0.920 respectively. The net monetary benefits of HS was higher than controls (US$ 92,118 vs US$ 91,650).

**Fig. 3:**
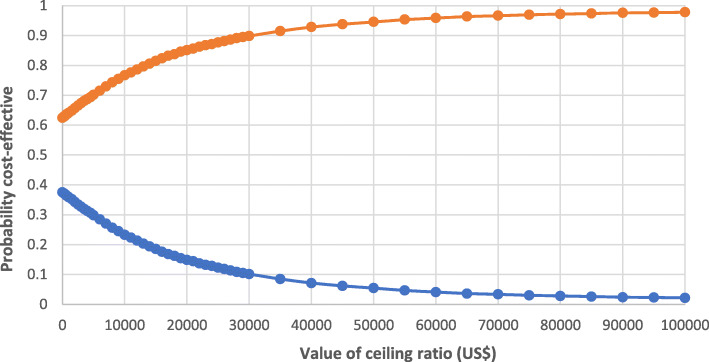
Cost-effectiveness acceptability curve

The Cost-effectiveness acceptability curve shows that the probability that HS provides a more cost-effective use of resources compared with standard therapy exceeds 85% (Fig. 3) using a Colombian thresholds of US$ 20.000 (threshold equivalent to three times the Colombian per-capita).

## Discussion

In this study, we evaluated the cost-effectiveness of HS inhalations compared to NS inhalations or no inhalations. Our study suggests that HS inhalations compared to control treatment, are unequivocally cost-effective (it is dominant, achieving better outcomes at lower cost). The HS inhalations reduce the cost per patient by around US $ 47 with a slight increase in the quality-adjusted life years. Although the magnitude of cost savings per patient is small, in a population perspective in which, for example in Colombia where more than 95.000 cases with acute respiratory infection in children under 2 years are hospitalized annually [25], the savings can be substantial for the health system.

The current evidence is conclusive regarding the reduction generated by the use of HS in the days of hospital stay. A recent meta-analysis of 23 RTCs shows that infants treated with 3% HS exhibited shorter durations of hospitalization compared with those treated with normal saline (NS; WMD = -0.43; 95% CI = -0.70, − 0.15) [8]. This intervention also has an important effect in reducing the probability of hospitalization, when used in the emergency department. Also, a recent meta-analysis of 18 RTCs, found a reduction of 23% in the probability of hospitalization (RR 0.771, 95% CI 0.619–0.959, I2 55.8%) [12]. Perhaps the lack of an economic and population perspective has led to variability in the recommendations regarding its routine use. For example, in some randomized clinical trials hypertonic saline has been a safe and effective intervention [31, 32], while other the hypertonic saline do not improves hospitalization rates or complications [33, 34]. A systematic review of guidelines of bronchiolitis, found also heterogeneous recommendations for the use of hypertonic. Seven guidelines did not recommend the use of hypertonic saline, whereas 8 in this review recommend its routine use in managing bronchiolitis [35]. Most of these clinical practice guidelines lack economic evaluations and are only limited to reviewing aspects of effectiveness and safety. Economic evaluations are key inputs for the generation of health technology recommendations, since they provide information on the efficiency of an intervention, such as inhalation HS, once it has already been shown to be effective and safe [36].

In previous economic evaluations cost savings have also been found for the use of HS inhalations in patients with bronchiolitis. A decision tree model developed by NICE (UK) in 2014, in their clinical practice guideline for bronchiolitis, found hypertonic saline would be cost-saving in 59% of simulations and when their only included recent evidence from clinical trials from 2010 de cost saving of HS increase to 76% [37]. A limitation of this model is that not including the probability of developing or not acute complications that do not require admission to intensive care, but that are managed during hospitalization. This can bias this model since the presence of acute complications is as frequent as 1 in 5 patients hospitalized with bronchiolitis [16]. This directly impacts both the costs and benefits of the model, underestimating the effect of interventions that prevent reaching this state such as HS nebulization. A recent cost-effectiveness evaluation in Finland by Heikkila et al., found a mean reduction in the hospitalization rate was 24%, and €146 ($199) savings per patient [10]. This economic evaluation used a structure of decision tree different from ours and had as outcome no QALYs as a benefit measure, but incremental cost-effectiveness ratio, savings per 1-h reduction in the LOS and incremental cost-effectiveness ratio and savings per 1% reduction in the hospitalization rate. A practical limitation of this study is that it separately evaluated the cost-effectiveness of HS nebulization in emergency and in another model in hospitalization. The scenario that is not real, in clinical scenario everything is a continuum, and there are summative effects in the total cost of HS in reducing the probability of hospitalization and in reducing LOS. Despite these limitations of previous studies, the fact that using a different model as this study in Finland and UK, savings have also been found using HS, with costs from developed countries, allows us to think that in a scenario with more limited resources but lower direct costs, such as in tropical countries, HS may be a solution for the efficient use of resources in a patient with bronchiolitis as we found in our study.

A very important aspect of our model is that it was robust to changing the values of the model’s utilities and costs. Regarding utilities, this result in the sensitivity analysis is important because they were extracted from the literature. Although they were collected from a European population, which may have different preferences for the health states evaluated, given their socio-economic and cultural context, our results did not change when exploring the change in the ICER in the range of values of each utility explored. The same happens with costs. Although the resources, frequencies of use, and costs were collected from tertiary centers, in Rionegro, and not from a national study with all hospitals in Colombia, modifications to their values in the sensitivity analysis also did not significantly change the ICER. These aspects give us confidence regarding the ability to make decisions with our results;, as is always necessary for science, more studies to replicate our results.

There are some limitations in this study. The cost data were from the years 2016 to 2017, and no further corrections were made. Our country has been characterized by having a very small price variation in the last 10 years, especially in health services between the different clinics and over time. Moreover, the proportions of each of the costs remain relatively constant, which, with few variations in their composition in the last ten years [38]. We use utilities extracted from the literature and not estimated directly from our population. As was mentioned previously, the reliability of the results was evaluated by sensitivity analyses.

## Conclusions

The HS was cost-effective in the inpatient treatment of infant bronchiolitis. Our study provides evidence that should be used by decision-makers to improve clinical practice guidelines and should be replicated to validate their results in other tropical countries.

## Data Availability

The raw data supporting your findings can be request to Jefferson.buendia@udea.edu.co

## Abbreviations

HS: Nebulized 3% hypertonic solution
QALYs: Quality-Adjusted Life-Years
PICU: Pediatric intensive critical unit
